# Comparison of digital and traditional skin wound closure assessment methods in mice

**DOI:** 10.1186/s42826-023-00176-1

**Published:** 2023-10-27

**Authors:** Coco X. Huang, Elisha Siwan, Sarah L. Fox, Matilda Longfield, Stephen M. Twigg, Danqing Min

**Affiliations:** 1 Greg Brown Diabetes and Endocrine Research Laboratory, Sydney Medical School (Central), Faculty of Medicine and Health, Charles Perkins Centre, The University of Sydney, Sydney, NSW Australia; 2 Department of Endocrinology, Royal Prince Alfred Hospital, Sydney, NSW Australia

**Keywords:** Digital imaging, Wound closure rate, Skin wound, Wound healing, Mouse model

## Abstract

**Background:**

Chronic skin wounds are a common complication of many diseases such as diabetes. Various traditional methods for assessing skin wound closure are used in animal studies, including wound tracing, calliper measurements and histological analysis. However, these methods have poorly defined wound closure or practical limitations. Digital image analysis of wounds is an increasingly popular, accessible alternative, but it is unclear whether digital assessment is consistent with traditional methods. This study aimed to optimise and compare digital wound closure assessment with traditional methods, using a diabetic mouse model. Diabetes was induced in male C57BL/6J mice by high-fat diet feeding combined with low dose (65 mg/kg of body weight) streptozotocin injections. Mice fed normal chow were included as controls. After 18 weeks, four circular full-thickness dorsal skin wounds of 4 mm diameter were created per mouse. The wounds were photographed and measured by callipers. Wound closure rate (WCR) was digitally assessed by two reporters using two methods: wound outline (WCR-O) and re-epithelialisation (WCR-E). Wounded skin tissues were collected at 10-days post-wounding and wound width was measured from haematoxylin and eosin-stained skin tissue.

**Results:**

Between reporters, WCR-O was more consistent than WCR-E, and WCR-O correlated with calliper measurements. Histological analysis supported digital assessments, especially WCR-E, when wounds were histologically closed.

**Conclusions:**

WCR-O could replace calliper measurements to measure skin wound closure, but WCR-E assessment requires further refinement. Small animal studies of skin wound healing can greatly benefit from standardised definitions of wound closure and more consistent digital assessment protocols.

**Supplementary Information:**

The online version contains supplementary material available at 10.1186/s42826-023-00176-1.

## Background

Impaired skin wound healing is a major cause of chronic wounds, which are a significant economic and health burden for individuals and healthcare systems [1]. People with diabetes and the elderly are at greater risk of developing chronic wounds, such as pressure sores and diabetic foot ulcers [2], which can significantly decrease their quality of life and lead to wound-related amputations, morbidity and mortality [1]. Although animal studies have been extensively used to investigate the pathophysiology of delayed skin wound healing [3–5], there are various existing methods for monitoring wound closure that each have their own challenges and may be incomparable between studies.

Traditional pre-clinical methods of examining skin wound healing include calliper measurements, *in-situ* wound tracing, and histological analysis [4]. Calliper measurements usually assume wounds are a certain shape and may not accurately represent wounds with irregular boundaries [6]. *In-situ* wound tracing, where wound edges are traced onto a transparent film, can account for irregular-shaped wounds [7]. However, this can be time-consuming for large wounds with complex topography and less reliable for smaller wounds; it may also disturb the wound and induce pain [7]. Both methods require animal anaesthetisation for accurate measurement, which raises ethical concerns when performed frequently. Histological analysis provides detailed information about wounds and skin structures [8] but requires animal euthanasia and larger sample sizes for temporal analysis. Altogether, these methods present various issues that need to be addressed for reliable skin wound closure analysis.

Digital imaging is a popular alternative that can overcome some of these challenges. Digital planimetry involves photographing a wound next to a ruler for calibration, positioning the camera lens perpendicular to the wound plane, then digitally identifying the wound area within the image using manual or computer-automated means [9]. This method is relatively inexpensive, reasonably accurate and precise [10], but there are varying definitions of wound closure in the literature and little consensus. Wounds may be considered closed if re-epithelialisation is evident without moist granulation tissue [11], whereas other studies measure open wounds by their outermost margin [12] or do not report their assessment method [13, 14]. Hence, there is a need for standardised, reliable, consistent and comparable digital wound assessment methods that can account for the complexities of healing and complement histological analysis.

This study aimed to analyse and optimise two methodologies of digital skin wound closure assessment and compare them to traditional methods in a murine model of diabetes, to determine their utility, reliability, and validity. We hypothesise that these digital techniques could be more appropriate and beneficial for analysing skin wound healing in animal research.

## Results

### Animal characteristics

**Fig. 1:**
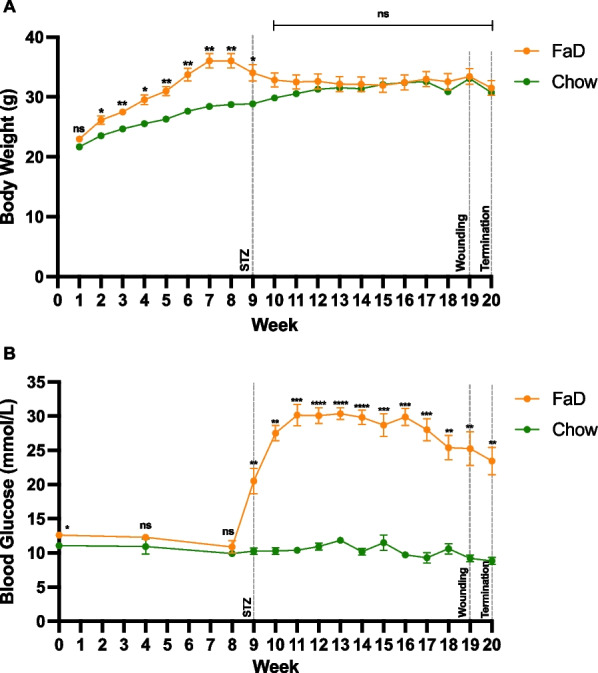
Mouse body weight (**A**) and random blood glucose (**B**) over the course of the study. FaD groups (n = 4) are shown in orange and Chow (n = 3) in green. Significance was indicated as follows: *p* < 0.05 (*), *p* < 0.01 (**), *p* < 0.001 (***), *p* < 0.0001 (****) and not significant (ns)

Pre-streptozotocin (STZ) administration, diabetic FaD mice were significantly heavier than control Chow (*p* < 0.05), except during the first week of the study (Fig. 1A). Post-STZ, their body weights did not significantly differ. FaD and Chow mice had similar blood glucose levels (BGL) pre-STZ administration, but afterwards, FaD consistently had higher BGL than Chow (*p* < 0.01), including at wounding and termination (Fig. 1B).

### Comparison of unblinded and blinded digital wound closure assessment

**Fig. 2:**
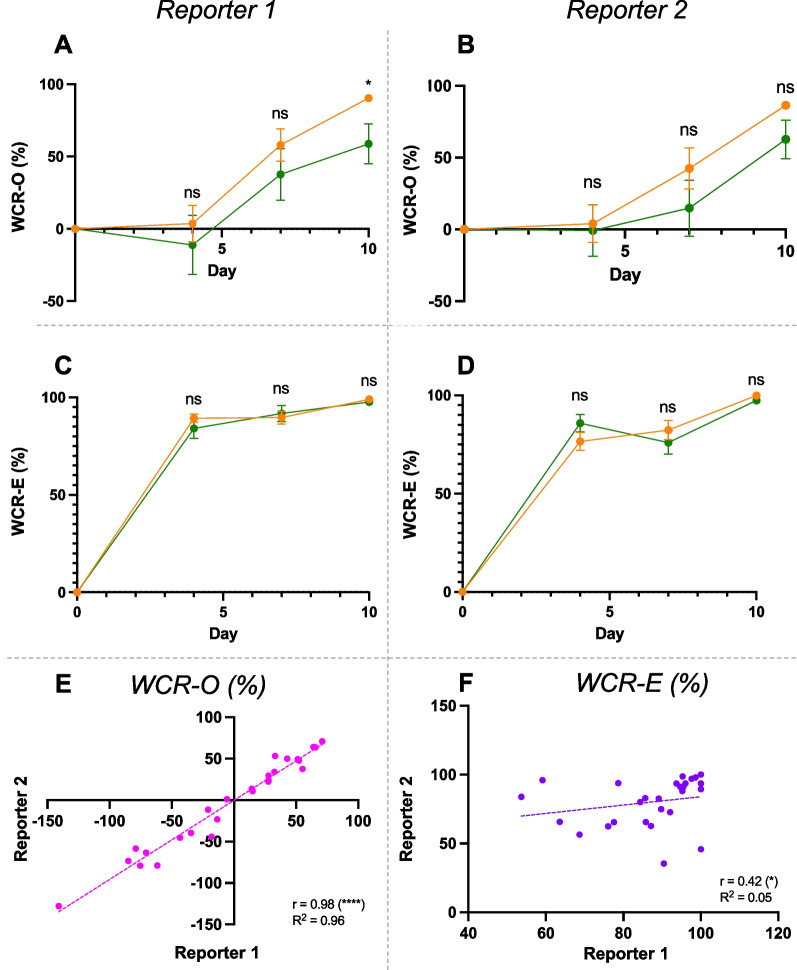
Reporters’ analysis of wound closure rate by wound outline (WCR-O) (**A**, **B**) and re-epithelialisation (WCR-E) (**C**, **D**). FaD groups (wound n = 16, 15, 15 and 5 at Day 0, 4, 7 and 10 respectively) are shown in orange and Chow (wound n = 12, 12, 11 and 7 at Day 0, 4, 7 and 10 respectively) in green. Linear regression was performed for WCR-O (**E**) and WCR-E (**F**) measurements between Reporter 1 (x-axis) and Reporter 2 (y-axis) at Day 4 (**E**, **F**). Correlation coefficient (r) with t-test and goodness of fit (R^2^) are presented. Reporter 1 was unblinded, Reporter 2 blinded. Significance was indicated as follows: *p* < 0.05 (*), *p* < 0.01 (**), *p* < 0.001 (***), *p* < 0.0001 (****)

At Day 10 post-wounding, digitally measured wound closure rate by wound outline (WCR-O) was significantly higher in FaD than Chow in Reporter 1’s assessment (FaD: 90.4 ± 1.7%; Chow: 58.9 ± 13.7%, *p* < 0.05) (Fig. 2A), but this trend was not significant in Reporter 2’s assessment (FaD: 86.4 ± 1.4%; Chow: 62.7 ± 13.4%, *p* = 0.11) (Fig. 2B). Digitally measured wound closure rate by re-epithelialisation (WCR-E) did not differ between FaD and Chow at all timepoints (Fig. 2C, D). Additionally, there was a strong positive linear correlation between the two reporters’ WCR-O (*p* < 0.0001) (Fig. 2E). WCR-E was less consistent between reporters, but their correlation was still significant (*p* < 0.05) (Fig. 2F).

### Comparison of digital and calliper wound closure assessment

**Fig. 3:**
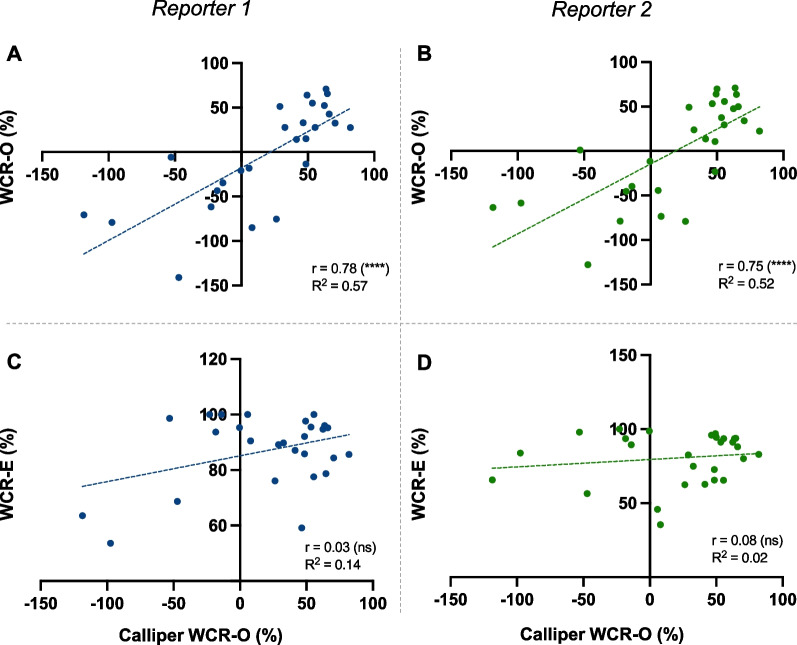
Linear regression comparing wound closure measured by callipers and digital methods at Day 4. Wound closure rate by wound outline (WCR-O) (**A**, **B**) and re-epithelialisation (WCR-E) (**C**, **D**) measured by digital imaging (y-axis) vs callipers (x-axis) for Reporter 1 (**A**, **C**) and Reporter 2 (**B**, **D**). Correlation coefficient (r) with t-test and goodness of fit (R^2^) are presented. Reporter 1 was unblinded, Reporter 2 blinded. Significance was indicated as follows: *p* < 0.05 (*), *p* < 0.01 (**), *p* < 0.001 (***), *p* < 0.0001 (****)

Both reporters found a strong positive linear correlation between calliper and digital WCR-O (*p* < 0.0001) (Fig. 3A, B). However, there was no correlation between calliper WCR-O and digital WCR-E (Fig. 3C, D). This finding was supported by the representative individual wound data at Day 4 post-wounding; digital and calliper WCR-O were mostly consistent, whereas digital WCR-E overestimated calliper WCR-O (Table 1).

**Table 1:** Comparison of calliper and digital measurements of wound closure at 4-days post-wounding

	Calliper WCR-O (%)^a^	Digital WCR-O (%)^b^		Digital WCR-E (%)^c^	
		Reporter 1^d^	Reporter 2^d^	Reporter 1	Reporter 2
Sample 1	− 0.3	− 20.7	− 11.5	95.3	98.7
Sample 2	28.7	25.2	30.8	82.4	95.1

^a^Calliper wound closure rate by wound outline

^b^Digital wound closure rate by wound outline

^c^Digital wound closure rate by re-epithelialisation

^d^Reporter 1 was unblinded, Reporter 2 blinded

### Histological assessment of wound closure

**Fig. 4:**
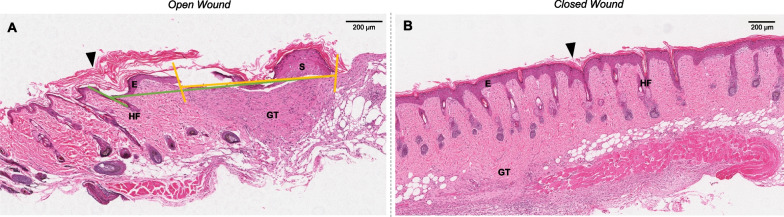
Histological wound closure assessment. Representative H&E staining for an open (**A**) and closed (**B**) wound at 10 days post-wounding. The green line represents total wound width; the yellow line represents open wound width. Wound closure rate by histological width (WCR-W) was expressed as a percentage of the total wound width that was not open. (E) Epidermis, containing epithelium; (GT) Granulation tissue; (HF) Hair follicle; (S) Scab; black arrowheads indicate loose keratin layers

Figure 4A, B shows representative wounds that were histologically open and closed respectively. Beneath scabs, the wounds were open as they had a discontinuous darker purple epithelium within the uppermost epidermal layer (Fig. 4A). Maturing granulation tissue beneath the open wound was visible, as well as loose keratin layers (arrowheads) on the wound surface, produced by maturing skin keratinocytes. In histologically closed wounds (Fig. 4B), a continuous dark purple epidermis and a much thinner, darker purple epithelium were visible on the most superficial surface, indicating complete re-epithelialisation. Some granulation tissue and loose keratin were also present (arrowheads).

At 10-days post-wounding, histological re-epithelialisation was expressed as wound closure rate by wound width (WCR-W), as shown in the same representative histologically open and closed wounds in Table 2. While Sample 1 was histologically open, this was inconsistent with digital WCR-E assessment; both reporters considered the wound to be over 95% closed by WCR-E, but histological assessment found only 31.7% of the wound had closed. WCR-O (~ 70%) was closer to the histological WCR-W (31.7%) but still did not accurately reflect histological wound closure as it indicated greater wound closure compared to WCR-W. Thus, both WCR-O and WCR-E overestimated the healing of histologically open wounds. However, for Sample 2, which was histologically closed, the digital measurements were mostly consistent with histological findings, especially the WCR-E.

**Table 2:** Comparison of histological and digital measurements of wound closure at 10-days post-wounding

	WCR-W (%)^a^	WCR-O (%)^b^		WCR-E (%)^c^	
		Reporter 1^d^	Reporter 2^d^	Reporter 1	Reporter 2
Sample 1	31.7	73.0	68.3	100.0	97.6
Sample 2	100.0	95.1	100.0	100.0	99.5

^a^Wound closure rate by wound width (histological measurement)

^b^Wound closure rate by wound outline (digital measurement)

^c^Wound closure rate by re-epithelialisation (digital measurement)

^d^Reporter 1 was unblinded, Reporter 2 blinded

## Discussion

Delayed wound healing causes a significant disease burden [15, 16]. Assessment of wound closure is fundamental to investigate impaired wound healing. In this study, two digital wound closure assessment methods were evaluated and compared with calliper and histological assessment. Digital wound assessment is user-friendly, accessible, and can be quickly performed on wounds over multiple timepoints without affecting the physical state of the wound or animal. It can also provide information on both WCR-O and WCR-E. We have shown that digital WCR-O has better inter-reporter reproducibility and more closely reflects calliper measurements of a wound.

At most timepoints, both reporters found no significant differences in either WCR-O or WCR-E between FaD and Chow, although FaD exhibited hyperglycaemia after STZ administration. This suggests the model was successful in inducing a diabetic phenotype in the mice, although delayed wound healing was not shown. Digital WCR-O was strongly positively and linearly correlated between reporters, indicating its reproducibility. Additionally, digital WCR-O showed a stronger positive, linear correlation with calliper WCR-O than calliper WCR-O with digital WCR-E, suggesting digital WCR-O can potentially replace calliper measurements for wound closure assessment.

WCR-E was less consistent between reporters due to the more subjective nature of judging re-epithelialisation. Clearer guidelines or higher quality wound images could be provided to aid judgement of WCR-E. Physically debriding scabs may provide clearer images of re-epithelialisation but could be a confounder, as it could reduce bacterial biofilms and benefit [14, 17] or disturb [18] wound healing. Ultrasonic techniques have been used to study deeper skin layers and measure wound depth as they can visualise underlying tissues even in the presence of scabs or other obstructions [19]. While identifying the uppermost epidermis and its layers by ultrasound is possible in pig skin [20], it may be more challenging in smaller animals such as mice. WCR-E may be easier to perform in clinical practice than animal studies as wounds can be monitored over a longer period, which may be sufficient for scabs to fall off. New technologies such as optical coherence tomography [21], which involves laser-imaging skin wounds and using deep machine learning to identify and monitor changes in epidermis and scab thickness, may also become viable methods to assess re-epithelialisation in the future when they become more accessible.

Calliper WCR-O is calculated from the length of the open wound and assumes wounds are circular and uniform in size. This is unrealistic as the wounds in this study varied in shape and size during healing. In contrast, digital WCR-O accounts for irregular wound outlines when determining the open wound area and reduces measurement error by directly tracing the outline. For accurate measurements using callipers, mice must also be stationary under anaesthesia, as their wounds may be too small to measure. However, anaesthetic procedures can affect the mouse and wound’s behavioural and biological functions if performed too frequently [22]. Cutting-edge 3D wound reconstruction and laser-scanner methods could more accurately capture the size and depth of small wounds [23, 24]. However, they would still require immobilisation by anaesthesia, are expensive and time-consuming, and may not be reliable for deep wounds covered by scabs [9, 25]. While these difficulties would not normally arise in a clinical setting, digital WCR-O assessment can overcome them when working with mice. Digital imaging allows data to be easily collected over multiple timepoints without requiring anaesthesia. Small wounds, especially in smaller animals such as mice, can also be traced more accurately by digital means, compared to calliper measurements and in-situ wound tracing.

However, as calliper and digital WCR-O do not account for the role of re-epithelialisation in wound healing, WCR-E or histological assessment are still necessary. Wound closure by re-epithelialisation is often inadequately described and defined [12–14]; re-epithelialisation was described in a splinted mouse skin model as “start[ing] from the wound edges and mov[ing] toward the center” [26], which is a broad definition that does not consider differences in wound colour and texture or scabs. When scabs are present, open wound area can be defined by the outer edge of a scab [11], but this interpretation could be too conservative if the scab remains over several days while re-epithelialisation occurs underneath. Thus, in this study, wound closure was judged digitally by wound outline and re-epithelialisation independently. These two methods allowed a comparison between the extent of wound re-epithelialisation and the area of underlying granulation tissue. This approach provides a more nuanced understanding of wound closure, avoiding reliance on a single, insufficient definition of wound closure.

The current gold standard to confirm wound closure is histological analysis, as it can give insights into re-epithelialisation and remodelling of underlying skin structures [8]. In histologically closed wounds, WCR-E represented this closure slightly more closely than WCR-O. However, WCR-O and WCR-E were less consistent with histologically open wounds and overestimated histological wound closure, especially WCR-E. These inconsistencies could be due to the presence of scabs obscuring the wound and limited sampling, as only one wound per mouse was histologically analysed and some mice had both open and closed wounds at termination. Additionally, histological WCR-W is a measure of distance, whereas WCR-O and WCR-E are measures of area, which could limit their comparability. As there was only one endpoint for skin sample collection at termination, it was not possible to histologically study wound closure at multiple timepoints in this study. Digital imaging can overcome these limitations and provide information on wound healing over multiple timepoints without affecting the physical state of the wound, which is another limitation of histological analysis in a clinical setting. However, histological assessment remains valuable for evaluating wound re-epithelialisation, especially at earlier timepoints, until more reliable digital methods are available.

Digital wound closure assessment can pose several challenges, such as inconsistent lighting, poor image resolution when magnified, and hair regrowth obscuring the wound. Optimising image brightness and contrast can help identify the wound outline, while shaving hair around wounds can improve clarity. Also, to prevent hair regrowth, wounding on black patches of shaved dorsal skin should be avoided. To prevent further loss of statistical power, WCR-O and WCR-E were not averaged for each mouse, although this would be ideal for better validity and consistency. Although mouse sample sizes were small, multiple wounds created per mouse enhanced the power of this study. Daily imaging was also helpful for monitoring and locating wounds, especially at later timepoints when they had almost completely healed. Future studies should consider more than one wound per mouse for histological analysis to improve validity. Additionally, we only assessed wound closure in male mice, whereas sex-based hormonal influences may contribute to wound healing rates and mechanisms [27]. However, digital wound closure assessment methods are expected to remain consistent regardless of the mice’s sex.

Future studies of wound closure by digital imaging should use a consistent source of lighting and a high-resolution camera to capture clearer images. WCR-O and WCR-E could also be digitally measured at earlier timepoints to clarify wound healing trends. Future reporters should be blinded to improve the validity of their WCR-O and WCR-E assessments and avoid bias, or machine learning techniques could be used to identify wound boundaries and re-epithelialisation. More work in optimising WCR-E assessment is needed, to standardise wound re-epithelialisation and improve inter-assessor reliability.

## Conclusions

In conclusion, current methods such as calliper measurements and histological analysis have several limitations, but digital imaging methods can overcome these as they are fast, inexpensive, non-invasive, and can track wound closure over several timepoints. Digital measurements of wound closure by wound outline strongly correlated with calliper measurements and could potentially replace them. However, further development is needed for digital assessment of wound closure by re-epithelialisation to enhance consistency among assessors and alignment with histological analysis. Histological assessment is still worthwhile where practical, as it provides structural information on skin wound healing where such information is otherwise unclear or difficult to obtain. The digital methods presented would be useful for future animal studies testing clinical or pharmaceutical wound healing interventions.

## Methods

### Animal model

Healthy male C57BL/6 J mice (n = 7) (Animal Resources Centre, Western Australia, Australia) were obtained at 5 weeks of age and then acclimatised for one week in individually ventilated cages, housing 3–4 mice per cage. The cages were furnished with enrichment bedding and nesting material and were maintained in a temperature and humidity-controlled environment with a 12-h light/12-h dark cycle at the University of Sydney’s Laboratory Animal Services. At 6 weeks old, the mice were randomly allocated into one of two groups: control or diabetes. Diabetes was induced using a modified method from our previous study [28]. Briefly, the diabetes group (FaD, n = 4) was fed an in-house high-fat diet containing 45% of calories from fat throughout the study period (Additional file 1A). After 8 weeks, the mice received intraperitoneal injections of STZ (Sigma-Aldrich, St. Louis, MO, USA) at 65 mg/kg of body weight, once daily for 2 days. The control group (Chow, n = 3) was fed a chow diet, containing 12% calories from fat (Specialty Feeds, Western Australia, Australia) (Additional file 1B). Mice were weighed weekly and random BGL was measured consistently in the morning by tail tip sampling using a blood glucose monitor (Abbott).

After 18 weeks, the mice were anaesthetised, and their dorsal fur was carefully removed by shaving and depilatory Nair™ Cream and cleaned with a saline-soaked gauze. After wiping dry their skin, skin antisepsis was performed by wiping the skin with chlorhexidine. Subsequently, four circular full-thickness dorsal skin wounds, each of 4 mm diameter, were induced per mouse by punch biopsy (Additional file 1C). The wounds were closely monitored and photographed with a smartphone camera immediately post-wounding and daily over the next 10 days. As adapted from a previous study [9], the camera was positioned perpendicular to the wound plane with a 10 cm ruler on the same flat surface as the mouse to account for differences in the height of the camera from the wounds. At 4-days post-wounding, calliper measurements of the wounds were taken under anaesthesia. At 10-days post-wounding, the mice were euthanised and skin surrounding each wound was collected for tissue analysis (Additional file 1D).

This study was approved by the University of Sydney Animal Ethics Committee (Project No.: 2020/1799). To comply with the principles of the 3Rs, the sample size was determined by considering 4 wounds per mouse to minimise the number of animals used, along with an additional 10% to account for potential exclusions due to STZ-induced effects. No incidents or wound infection were observed during the study period. Surgery was performed under inhaled isoflurane anaesthesia, and buprenorphine (Temgesic, Sigma-Aldrich, St. Louis, MO, USA) at a dosage of 0.1 mg/kg of body weight was administered subcutaneously for pre-surgery and continued twice daily (every 12 h) post-surgery for pain management. All efforts were made to minimise suffering, as per the Animal Research Act 1985, Animal Research Regulation 2021, and Australian code for the care and use of animals for scientific purposes (updated 2021) (Australia).

### Calliper assessment of wound area

Calliper assessment was performed under anaesthesia at 4-days post-wounding by an unblinded assessor. This assessment was also intended to be performed under anaesthesia at 7- and 10-days post-wounding but did not occur as the wounds were too small to be measured by callipers at these timepoints. For each wound, three calliper measurements were taken: vertically, horizontally, and diagonally across the wound. These diameter measurements were averaged, and wound area was calculated using the area of a circle formula. Initial wound area was expressed as the area of a circle of 4 mm diameter.

### Digital assessment of wound area

Wound area was quantified by digital analysis of photos taken at 0-, 4-, 7- and 10-days post-wounding using ImageJ (NIH, v.2.1.0/1.53c). Wound area was digitally traced and measured using two methods (Additional file 2A). To calculate the wound closure rate (WCR) by wound outline (WCR-O), the outermost edge of the wound was traced. This value considers underlying wound tissues that may still be remodelling after a scab formed, even after the regrowth of the top epithelial layer of skin [2]. Wound closure rate by re-epithelialisation (WCR-E) was measured by digitally tracing the wound area that had not yet re-epithelialised. The border between moist, open wound and dry surrounding tissue was used to judge re-epithelialisation [11]. Where this was unclear due to scabbing, darker areas within scabs were treated as “open” wound; otherwise, uniformly dark red and flaking scabs were treated as completely closed (Additional file 2B). Poor quality images of wounds, including those heavily covered by hair or fused together, were excluded when encountered. Wound tracing was performed by two independent reporters who were either unblinded (Reporter 1) or blinded to the identity of the mice (Reporter 2). They used a standardised protocol with guideline images to aid their judgement of wound closure (Additional file 2B).

### Wound closure rate calculation

Digital WCR-O, WCR-E and calliper WCR-O were expressed as the percentage of the initial wound area (Day 0) that had closed at each timepoint:

$$ WCR - O~\,or\,~WCR{-}E~(\% ) = \frac{{Day\,0\,wound\,area - ~Day\,X\,~wound\,area}}{{Day\,0\,wound\,area}}~ \times ~100 $$

WCR-O and WCR-E at Day 0 were set as 0%.

### Histological assessment

Histological analysis of wound skin tissues collected at 10-days post-wounding was performed unblinded using haematoxylin and eosin (H&E) staining on 5 µm sections, imaged under 400X magnification. The wounded area was identified between the epithelial tongues [29] and a green line was drawn at the outermost edge of each epithelial tongue flanking the wound (Fig. 4A). An additional line was drawn between where each green line intersected the epithelium, and this distance was taken as the total wound width. This process was repeated for the innermost tips of the epithelial tongues to identify the open wound width, shown by the yellow line. Histological wound width closure (WCR-W) at Day 10 was expressed as a percentage of the wound width that was closed, compared to the total wound width. Picrosirius red-stained images were used to help identify the wound boundaries (data not included).

### Statistical analysis

All statistical tests were performed using GraphPad Prism (v.9.0.2). Statistical significance was accepted at *p* < 0.05. Body weight, BGL, WCR-O and WCR-E were analysed between FaD and Chow. Normality testing was performed using the D’Agostino-Pearson or Shapiro–Wilk test. Parametric data was analysed with a two-tailed Student’s t-test with Welch’s correction and reported as the mean ± standard error, while non-parametric data was analysed with a two-tailed Mann–Whitney U test and reported as the median and interquartile range.

Simple linear regression was performed to determine the correlation between WCR calculated by callipers and digital methods at Day 4. Pearson and Spearman correlation coefficients were calculated for parametric and non-parametric data respectively, along with *p*-values and goodness of fit (R^2^).

### Supplementary Information

**Additional file 1**. Mouse diabetes induction and wounding. At the age of 6 weeks, C57BL/6J mice were either (**A**) fed a high-fat diet and received low-dose streptozotocin (STZ) injections to induce diabetes (FaD, mouse n=4) or (**B**) fed normal chow (mouse n=3). (**C**) After 18 weeks, wounding occurred. (**D**) Wounds were photographed daily for the next 10 days, then the mice were euthanised. Wounded skin samples were collected at (**C**) wounding and (**D**) termination.

**Additional file 2**. Digital wound closure assessment methods. (**A**) Example of digital wound closure assessment by wound outline (WCR-O) and re-epithelialisation (WCR-E). Images of the same wound at 0-, 4-, 7- and 10-days post-wounding were digitally traced (yellow line) using ImageJ. (**B**) Assessment of scabbed wounds by WCR-O and WCR-E. Each column displays the same wound, and the yellow line indicates the traced perimeter. Colour and contrast enhancement was occasionally used to distinguish darker, open areas (WCR-E, central image).

## Data Availability

All data generated or analysed during this study are available from the corresponding author upon reasonable request.

## Abbreviations

BGL: Blood glucose level
Chow: Chow-fed mice
FaD: Diabetic mice (high-fat diet fed with STZ injections)
STZ: Streptozotocin
WCR: Wound closure rate
WCR-E: Wound closure rate by re-epithelialisation
WCR-O: Wound closure rate by wound outline
WCR-W: Wound closure rate by histological width
