# Supplementary material for: Comparison of digital and traditional skin wound closure assessment methods in mice
Source: Lab Anim Res. 2023 Oct 27;39:25. doi: 10.1186/s42826-023-00176-1 (PMC10605778; doi:10.1186/s42826-023-00176-1)
Supplement: Supplementary file 1 — Additional file 1. Mouse diabetes induction and wounding. At the age of 6 weeks, C57BL/6J mice were either (A) fed a high-fat diet and received low-dose streptozotocin (STZ) injections to induce diabetes (FaD, mouse n=4) or (B) fed normal chow (mouse n=3). (C) After 18 weeks, wounding occurred. (D) Wounds were photographed daily for the next 10 days, then the mice were euthanised. Wounded skin samples were collected at (C) wounding and (D) termination. [file 42826_2023_176_MOESM1_ESM.pptx]

## Slide 1
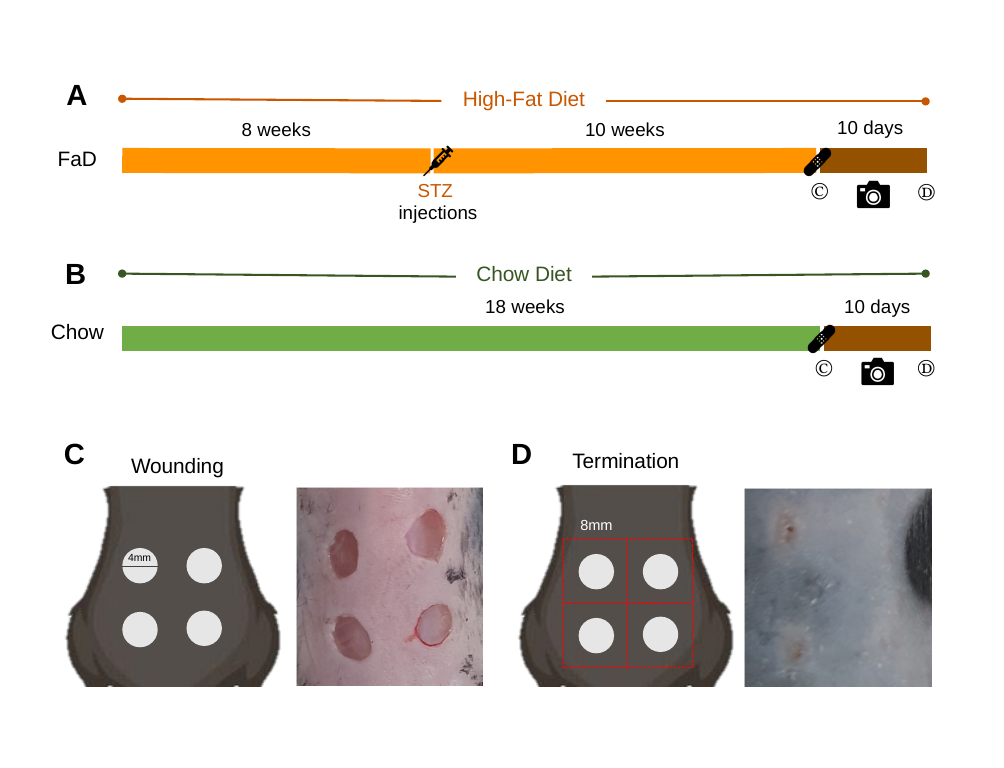

A
High-Fat Diet
10 days
10 weeks
8 weeks
STZ
injections
Ⓒ
Ⓓ
FaD
Chow Diet
18 weeks
10 days
B
Chow
Ⓒ
Ⓓ
D
C
Termination
Wounding
8mm
4mm
