# Supplementary figures and images for: Comparison of digital and traditional skin wound closure assessment methods in mice
Source: Lab Anim Res. 2023 Oct 27;39:25. doi: 10.1186/s42826-023-00176-1 (PMC10605778; doi:10.1186/s42826-023-00176-1)

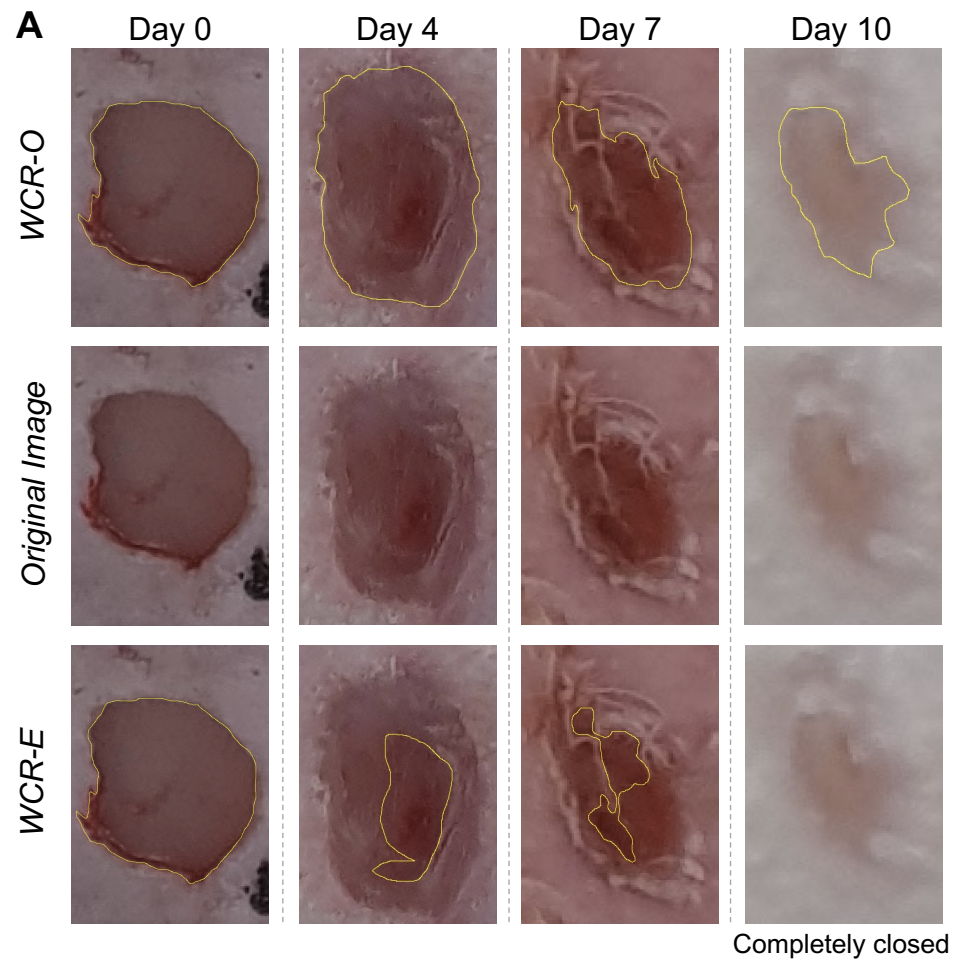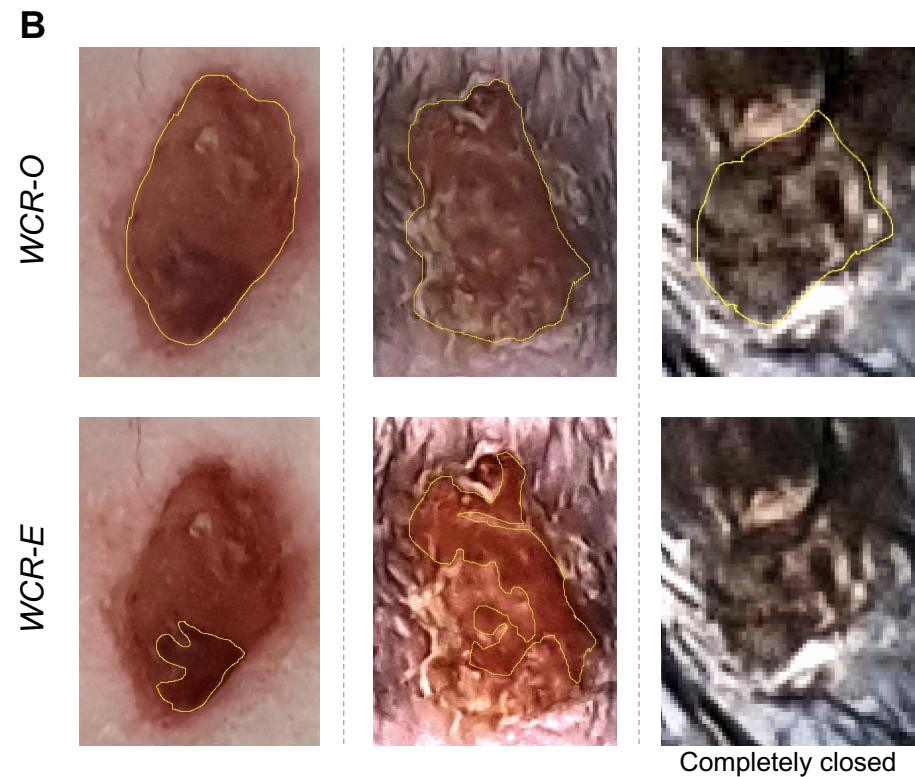

Supplement: Supplementary file 2 — Additional file 2. Digital wound closure assessment methods. (A) Example of digital wound closure assessment by wound outline (WCR-O) and re-epithelialisation (WCR-E). Images of the same wound at 0-, 4-, 7- and 10-days post-wounding were digitally traced (yellow line) using ImageJ. (B) Assessment of scabbed wounds by WCR-O and WCR-E. Each column displays the same wound, and the yellow line indicates the traced perimeter. Colour and contrast enhancement was occasionally used to distinguish darker, open areas (WCR-E, central image). [file 42826_2023_176_MOESM2_ESM.pdf]
